# Implementation interventions in preventing surgical site infections in abdominal surgery: a systematic review

**DOI:** 10.1186/s12913-020-4995-z

**Published:** 2020-03-20

**Authors:** Ivonne Tomsic, Nicole R. Heinze, Iris F. Chaberny, Christian Krauth, Bettina Schock, Thomas von Lengerke

**Affiliations:** 1 Hannover Medical School, Centre for Public Health and Healthcare, Department of Medical Psychology, Carl-Neuberg-Str. 1, 30625 Hannover, Germany; 2 Hannover Medical School, Centre for Public Health and Healthcare, Institute of Epidemiology, Social Medicine and Health Systems Research, Carl-Neuberg-Str. 1, 30625 Hannover, Germany; 3 Leipzig University Hospital, Centre for Infection Medicine (ZINF), Institute of Hygiene, Hospital Epidemiology and Environmental Medicine, Liebigstr. 22, 04103 Leipzig, Germany

**Keywords:** Surgical site infection prevention, Implementation intervention, Guideline dissemination, Professional compliance, Abdominal surgery

## Abstract

**Background:**

Surgical site infections (SSIs) are highly prevalent in abdominal surgery despite evidence-based prevention measures. Since guidelines are not self-implementing and SSI-preventive compliance is often insufficient, implementation interventions have been developed to promote compliance. This systematic review aims to identify implementation interventions used in abdominal surgery to prevent SSIs and determine associations with SSI reductions.

**Methods:**

Literature was searched in April 2018 (Medline/PubMed and Web of Science Core Collection). Implementation interventions were classified using the implementation subcategories of the EPOC Taxonomy (Cochrane Review Group Effective Practice and Organisation of Care, EPOC). Additionally, an effectiveness analysis was conducted on the association between the number of implementation interventions, specific compositions thereof, and absolute and relative SSI risk reductions.

**Results:**

Forty studies were included. Implementation interventions used most frequently (“top five”) were audit and feedback (80% of studies), organizational culture (70%), monitoring the performance of healthcare delivery (65%), reminders (53%), and educational meetings (45%). Twenty-nine studies (72.5%) used a multimodal strategy (≥3 interventions). An effectiveness analysis revealed significant absolute and relative SSI risk reductions. E.g., numerically, the largest absolute risk reduction of 10.8% pertained to thirteen studies using 3–5 interventions (*p* < .001); however, this was from a higher baseline rate than those with fewer or more interventions. The largest relative risk reduction was 52.4% for studies employing the top five interventions, compared to 43.1% for those not including these. Furthermore, neither the differences in risk reduction between studies with different numbers of implementation interventions (bundle size) nor between studies including the top five interventions (vs. not) were significant.

**Conclusion:**

In SSI prevention in abdominal surgery, mostly standard bundles of implementation interventions are applied. While an effectiveness analysis of differences in SSI risk reduction by number and type of interventions did not render conclusive results, use of standard interventions such as audit and feedback, organizational culture, monitoring, reminders, and education at least does not seem to represent preventive malpractice. Further research should determine implementation interventions, or bundles thereof, which are most effective in promoting compliance with SSI-preventive measures in abdominal surgery.

## Background

Surgical site infections (SSIs) are among the most common healthcare-associated infections in Europe [1]. In 2016, 22.4% of all reported healthcare-associated infections in Germany were SSIs [2]. In abdominal surgery, SSI rates are particularly high [3, 4]. For example, the SSI rate in colon surgery, as reported by the European Centre for Disease Prevention and Control (ECDC) for 2016, was 9% across 12 European countries, with a range from 5.3 to 18% [5]. Regarding their effects, SSIs can negatively impact patients and their families by increasing morbidity as well as mortality, causing additional healthcare costs by extending the length of hospital stay, and increasing the need for cost-intensive treatments [6–11]. Thus, SSIs represent a significant burden and challenge for healthcare systems and institutions.

Regarding the prevention of SSIs, several guidelines have been published in which a variety of evidence-based measures are recommended, e.g., the “Global Guidelines for the Prevention of Surgical Site Infection” by the World Health Organization (WHO) [12]. In colorectal surgery, for instance, a recent meta-analysis has shown that preventive measures, when used in the form of bundles (i.e., sets of usually three to five measures implemented in a combined and consistent fashion [13]) reduce SSI risk by an average of 40.2% [14].

At the same time, guidelines are not self-implementing. That is, the implementation of measures specified in guidelines—and thus complying with these recommendations—is often challenging for various reasons, most notably because of internal and external barriers. While internal barriers mostly relate to personal factors such as lack of knowledge or low motivation, external barriers refer to environmental factors such as missing equipment or lack of leadership [15, 16]. This also holds true for SSI prevention, and compliance with measures to prevent SSIs is often suboptimal. For instance, Leaper and colleagues have reported that studies in the United States (US) and the United Kingdom (UK) show compliance rates ranging from 20 to 60% [17]. Another systematic review has revealed that compliance with adequate antibiotic prophylaxis ranges from 0.3 to 84.5% across thirteen studies and that seven of these studies report rates of less than 50% [18].

**Fig. 1:**
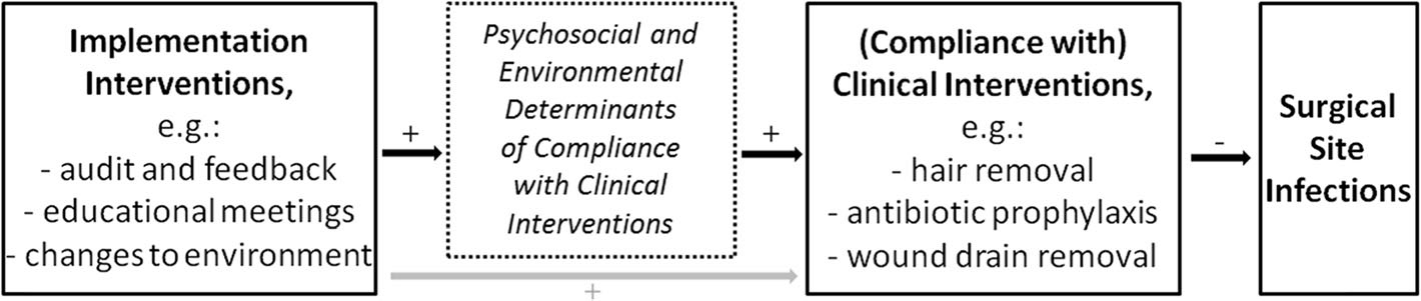
Schematic representation of two types of interventions to prevent surgical site infections

The question of how to overcome barriers to guideline implementation by promoting compliance with recommended measures and thus translate evidence into practice has been increasingly addressed by the evolving field of implementation research [19]. One concept that has been proposed in this context is that of implementation interventions. An implementation intervention has been defined as a “… method or technique designed to enhance adoption of a “clinical” intervention …” [20] (p. 218), in which context a clinical intervention refers to a “… specific clinical/therapeutic practice … , or delivery system/organizational arrangement … , or health promotion activity … being tested or implemented to improve health care outcomes” [20] (p. 218). Correspondingly, in prevention, clinical interventions refer to measures that prevent the disease in question in a comparatively direct manner. For SSI prevention, Fig. 1 shows these links between implementation and clinical interventions. Measures such as hair removal, antibiotic prophylaxis and wound drain removal have a rather immediate preventive effect on SSIs and thus are classified as “clinical interventions”. In contrast, while implementation interventions such as audit and feedback, educational measures and environmental changes may also prevent SSIs, they do so indirectly via their effects on compliance with clinical interventions. Usually, this effect (depicted by the grey arrow in Fig. 1) is mediated by the effects of the implementation interventions on the psychosocial or environmental determinants of compliance (black arrows with “+”-indication). For instance, an educational meeting on hand hygiene usually aims at improving healthcare workers’ knowledge and motivation related to the clinical intervention of “hand hygiene”, which then influences the behaviour in terms of guideline compliance. Changing a ward environment in terms of providing disinfectant dispensers at identified optimal points-of-care represents another example of an implementation intervention, which is directed at an environmental determinant of compliant hand hygiene behaviour.

A large variety of implementation interventions exist that can potentially be used to promote compliance with guidelines. For infection prevention in general, the WHO has published the “Guidelines on Core Components of Infection Prevention and Control Programmes at the National and Acute Health Care Facility Level” [21, 22]. Here, the WHO recommends the use of multimodal strategies that contain three or more (usually five) different types of implementation interventions to facilitate infection prevention and control activities [21, 22].

Regarding SSI prevention in particular, Ariyo and colleagues have conducted a systematic review on utilized implementation strategies [23]. They categorize implementation interventions using the “four Es” approach [24, 25], which describes “engage, “educate”, “execute” and “evaluate” as the basic components of change management. That is, this approach serves as a classification system for more tangible implementation interventions. The review identifies a multitude of effective implementation interventions, such as multidisciplinary teams, leadership involvement, staff education, checklists and reminders, and monitoring and feedback. However, one limitation of the study was that it did not differentiate between surgical specialties such as abdominal and orthopaedic surgery. Thus, these potential differences remained unidentified. Furthermore, the study did not report the differences regarding SSI-preventive effects pooled across studies. Finally, out of the different classification systems developed to categorize implementation interventions [24–27], the study used the “four Es” approach, which, while being concise, represents a rather coarse-grained system with four relatively general categories.

Against this background, and especially considering the high SSI rates in abdominal surgery as previously noted, this systematic review aims to give an account of what has actually been carried out in the field of abdominal surgery thus far in terms of implementation interventions to prevent SSIs and to what effect. To present identified implementation interventions, the EPOC Taxonomy developed for health system interventions by the Cochrane Review Group Effective Practice and Organisation of Care (EPOC) [26] will be used. Having been successfully used in earlier reviews [28, 29], this taxonomy allows to describe implementation studies not only in a unified fashion but also in a manner more detailed than the “four Es” approach, thus facilitating comparisons. For present purposes, twenty subcategories of the taxonomy that describe implementation interventions and focus on healthcare organizations and the behaviour of healthcare professionals will be employed. Furthermore, the review will strive to determine associations in abdominal surgery between implementation interventions on the one hand and compliance with clinical interventions and reductions in SSI rates on the other hand.

## Materials and methods

This manuscript conforms to the Preferred Reporting Items for Systematic Reviews and Meta-Analysis (PRISMA) Statement [30].

### Search strategy

The literature search was conducted on April 27, 2018 in the Medline/PubMed and Web of Science Core Collection databases. Studies that were published before January 1, 2018 were included. In the search strategy, key topics were combined (abdominal surgery, surgical site infections, guideline implementation/guideline compliance, and implementation interventions). For each topic, related essential terms were taken into account. For the detailed search strategy, see Additional file 1.

### Study selection

Two reviewers (IT & NRH) screened the records independently. In cases of discrepancies during the screening of titles, the studies were included in the abstract screening. When there was a discrepancy during the abstract screening, the study was included in the full-text screening. When there was a discrepancy regarding the inclusion of a study during the full text screening, the reviewers discussed the study until a consensus was reached. No third reviewer was needed.

Studies of all design types were included, whereas research articles such as editorials, letters, commentaries, abstracts or protocols were excluded. Studies were included if they focused on the prevention of SSIs in abdominal surgery and if implementation interventions were applied and reported. Studies that focused on more than one surgery field were included if abdominal surgery was one of the fields. Studies that only focused on another surgery field, such as gynaecology or urology, were excluded. Studies were included when the implementation interventions were used to promote compliance in healthcare workers. Studies that exclusively focused on patient compliance were excluded. Only full texts in the English or German language were included.

### Data extraction and quality assessment

For the extraction process, an extraction table was created that contained the following topics: author, publication year, country, study design, specific type of surgery, used implementation interventions, baseline and cohort sample sizes, baseline and cohort outcomes, baseline and cohort SSI rates, and baseline and cohort compliance rates. The extraction of the data was performed by two reviewers (IT & NRH). In accordance with the predominant design of the studies, the “Quality Assessment Tool for Before-After (Pre-Post) Studies With No Control Group” developed by the National Heart, Lung and Blood Institute was used for quality assessment (risk of bias) [31]. This tool has already been used successfully in earlier studies [32, 33].

### Implementation intervention classification

From the EPOC Taxonomy’s domain of “Implementation Strategies” [26], which had been chosen as the system by which to classify implementation interventions (see the Background section), those categories targeting healthcare organizations and the behaviour of healthcare workers were considered, whereas those targeting healthcare recipients were not. While being designated as intervention strategies in the EPOC Taxonomy, single EPOC categories are hereafter (in accordance with [20]; see the Background section) referred to as implementation interventions, while the term “implementation strategy” is used to refer to a set in terms of a ““bundle” of implementation interventions” [20] (p. 218). Table 1 shows the 20 EPOC categories used, together with their definitions [26] and typical examples. The extracted implementation interventions from the included studies were classified independently into the EPOC category system by two reviewers (IT & TvL). When discrepancies were encountered, the reviewers discussed them until an agreement was reached. When different implementation interventions were used that fit into one and the same EPOC category, this category was rated only once. When an implementation intervention fit into two or more categories, all relevant categories were coded.

**Table 1:** EPOC implementation subcategories and definitions [26] with examples

EPOC implementation subcategory	Definition^a^	Examples
Organisational culture	Strategies to change organisational culture	Multidisciplinary teams, steering committees, regular briefings, leadership/leaders’ involvement
Audit and feedback	A summary of health workers’ performance over a specified period of time, given to them in a written, electronic or verbal format. The summary may include recommendations for clinical action	Feedback sessions, personal performance feedback, posting SSI or compliance rates
Clinical incident reporting	System for reporting critical incidents	Critical Incident Reporting Systems
Monitoring the performance of the delivery of healthcare	Monitoring of health services by individuals or healthcare organisations, for example by comparing with an external standard	Monitoring compliance with SSI preventive measures, monitoring SSI incidence
Communities of practice	Groups of people with a common interest who deepen their knowledge and expertise in this area by interacting on an ongoing basis	Regional hospital collaboration to regularly exchange knowledge and improve quality
Continuous quality improvement	An iterative process to review and improve care that includes involvement of healthcare teams, analysis of a process or system, a structured process improvement method or problem solving approach, and use of data analysis to assess changes	Regular meetings to review compliance with preventive measures and when necessary to eliminate barriers to improve quality of care
Educational games	The use of games as an educational strategy to improve standards of care	Video games, smartphone-based games, quizzes
Educational materials	Distribution to individuals, or groups, of educational materials to support clinical care, i.e., any intervention in which knowledge is distributed. For example this may be facilitated by the internet, learning critical appraisal skills; skills for electronic retrieval of information, diagnostic formulation; question formulation	Posters, newsletters, bulletins
Educational meetings	Courses, workshops, conferences or other educational meetings	Educational sessions, educational lectures, grand round lectures, workshops
Educational outreach visits, or academic detailing	Personal visits by a trained person to health workers in their own settings, to provide information with the aim of changing practice	Site visits by a trained healthcare professional to educate groups or individuals
Clinical Practice Guidelines	Clinical guidelines are systematically developed statements to assist healthcare providers and patients to decide on appropriate health care for specific clinical circumstances’(US IOM)	Developing a new clinical practice guideline, choosing evidence-based guidelines
Inter-professional education	Continuing education for health professionals that involves more than one profession in joint, interactive learning	Interdisciplinary education
Local consensus processes	Formal or informal local consensus processes, for example agreeing a clinical protocol to manage a patient group, adapting a guideline for a local health system or promoting the implementation of guidelines	Clinical practice guideline development with agreeing from all levels
Local opinion leaders	The identification and use of identifiable local opinion leaders to promote good clinical practice	Involvement of project officers, study champions
Managerial supervision	Routine supervision visits by health staff	Supervision by managerial staff
Patient-mediated interventions	Any intervention aimed at changing the performance of healthcare professionals through interactions with patients, or information provided by or to patients	Patient feedback, patients as committee members
Public release of performance data	Informing the public about healthcare providers by the release of performance data in written or electronic form.	Publicly accessible websites that provide performance reports
Reminders	Manual or computerised interventions that prompt health workers to perform an action during a consultation with a patient, for example computer decision support systems	Checklists, automatic electronic reminders, protocols
Routine patient-reported outcome measures	Routine administration and reporting of patient-reported outcome measures to providers and/or patients	Assessing patients’ experience of symptoms through questionnaires before and after interventions
Tailored interventions	Interventions to change practice that are selected based on an assessment of barriers to change, for example through interviews or surveys	Developing implementation interventions based on previously identified barriers

^a^ Note: This column shows the original definitions as presented in [26]

### Effectiveness analysis

Regarding the effectiveness of implementation interventions, first, the association of the number of implementation interventions in studies with their achieved SSI rate reduction was analysed. Given the definition of multimodal strategies (three or more and usually five [22]), studies were grouped as follows: 1–2 vs. 3–5 vs. 6 or more implementation interventions. Second, studies including the five, four or three most frequent implementation interventions were compared to those where this did not apply, i.e., in which not all of these interventions were included. In both cases, a general linear model (GLM) repeated measures analysis was conducted using IBM SPSS® Statistics 25. For each group of studies as defined, the mean value of the baseline and cohort SSI rates and the mean value of the absolute and relative SSI risk difference were calculated. Subsequently, the differences in the baseline and cohort SSI rates between groups with either different numbers of implementation interventions or including (vs. not including) the most frequent interventions were tested. Additionally, tests of differences across the baseline and cohort SSI rates within all of these groups were performed.

## Results

### Review statistics

**Fig. 2:**
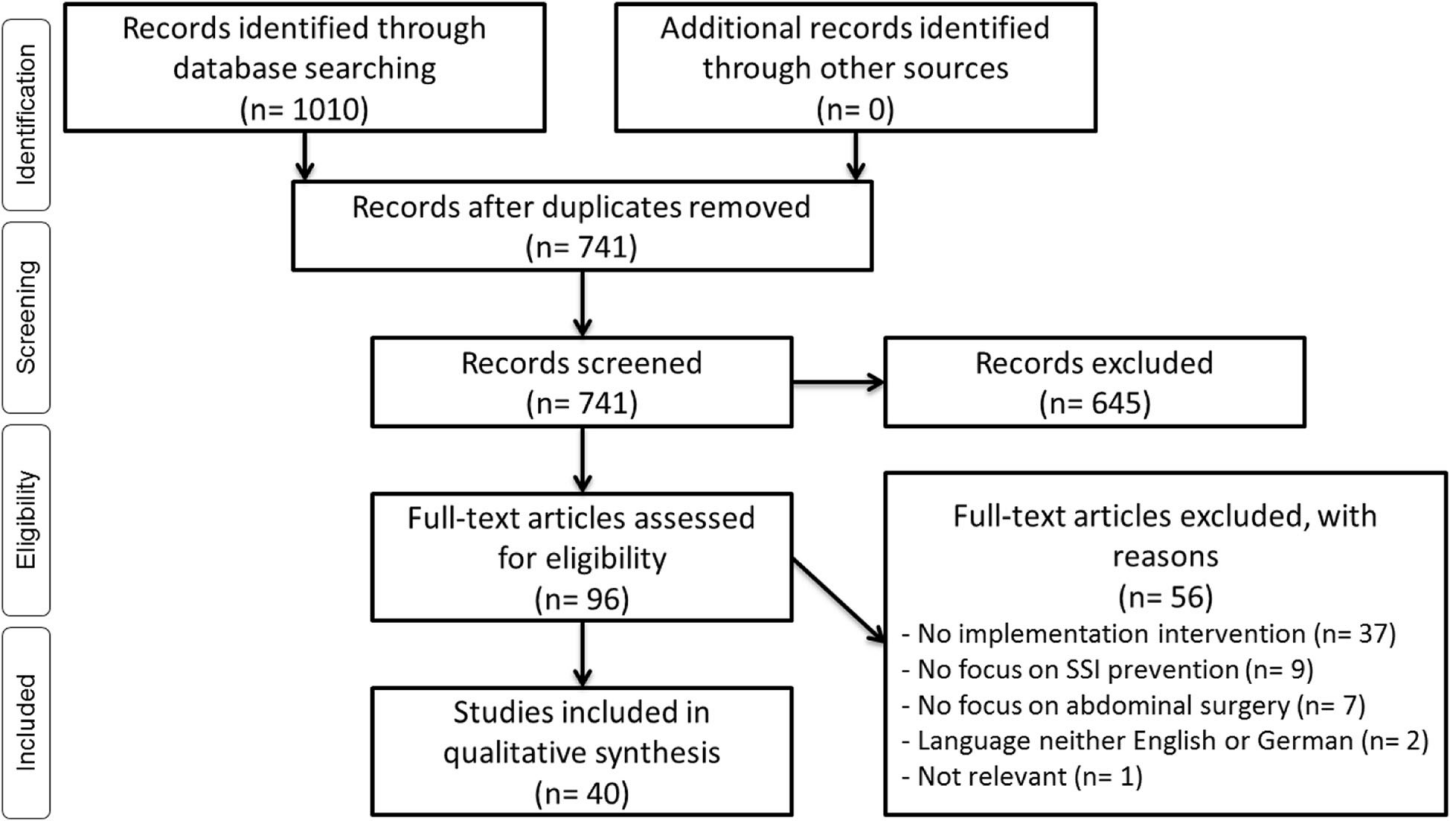
PRISMA flow diagram of the study selection process (following Moher et al. [30])

Through the literature search, 1010 records were identified. The review process is presented in a PRISMA flow diagram [30] in Fig. 2. After duplicates were removed, 741 publications remained for title screening. After title and abstract screening, 96 studies remained for full text screening. Finally, 40 studies met the inclusion criteria and were included in the qualitative synthesis.

### Characteristics of the included studies

The main characteristics of all 40 included studies are presented in Table 2. Twenty-five studies were performed in the US [36, 37, 39, 45–52, 55, 56, 58–60, 62, 63, 65, 67, 69–73], two each in Australia [34, 54], Canada [41, 42], the Netherlands [38, 44], Spain [40, 64], and the UK [35, 66], and one each in Germany [68], Saudi Arabia [53], Singapore [57], Switzerland [61], and Qatar [43]. Thirty-six studies were single-center studies, and four studies were performed in more than one hospital [50, 65, 66, 69]. More than half of the included studies (*n* = 22) were published after 2014. In 2017, the most studies were published (*n* = 7), while the oldest study dated back to 2004. All included studies were cohort studies, and 38 of them were uncontrolled before-after studies. Thirty-six studies exclusively dealt with abdominal surgery, and 23 of these focused on colorectal surgery. There were four studies that also considered other surgery fields in addition to abdominal surgery [50, 58, 61, 67]. The number of clinical interventions varied across the studies. Thirty-four studies targeted a bundle of clinical interventions, while six studies focused on antibiotic prophylaxis only and analysed this intervention regarding specific attributes (e.g., timing, selection and dose; see Additional file 2).

**Table 2:** Study characteristics of *N* = 40 included studies

First author, year, country	Study design	Type of surgery	Sample size	SSI Outcome	SSI rate	Compliance rate
Bull, 2011, Australia [34]	Cohort (before/after)	Colorectal surgery	Baseline: NR Cohort: 142	Baseline: NR Cohort: 10	Baseline: 15% Cohort: 7%	Baseline: 5,3% (global) Cohort: 21,1% (global)
Cameron, 2015, UK^c^ [35]	Cohort (before/after)	Gastrointestinal surgery	Baseline: 58 Cohort: 73	Baseline: NR Cohort: NR	Baseline: NR Cohort: NR	Baseline: 31% (global) Cohort: 73% (global)
Cima, 2013, US [36]	Cohort (before/after)	Colorectal surgery	Baseline: 531 Cohort: 198	Baseline: 52 Cohort: 8	Baseline: 9.8% Cohort: 4%	Baseline: only for individual measures Cohort: only for individual measures
Connolly, 2016, US [37]	Cohort (before/after)	Colorectal surgery	Baseline: 379 Cohort: 328	Baseline: 122 Cohort: 27	Baseline: 32.2% Cohort: 8.2%	Baseline: NR Cohort: NR
Crolla, 2012, Netherlands [38]	Cohort (before/after)	Colorectal surgery	Baseline: 349 Cohort: 377	Baseline: 85 Cohort: 61	Baseline: 21.6% Cohort: 16.2%	Baseline: 10% (global) Cohort: 80% (global)
DeHaas, 2016, US [39]	Cohort (before/after)	Colorectal surgery	Baseline: 277 Cohort: 254	Baseline: 49^a^ Cohort: 13^a^	Baseline: 17.58% Cohort: 5.11%	Baseline: NR Cohort: NR
Elia-Guedea, 2017, Spain [40]	Cohort (before/after)	Colorectal surgery	Baseline: 70 Cohort: 79	Baseline: 22 Cohort: 11	Baseline: 31.4% Cohort: 13.9%	Baseline: NR Cohort: NR
Forbes, 2008, Canada^c^ [41]	Cohort (before/after)	Colorectal or hepatobiliary surgery	Baseline: 105 Cohort: 103	Baseline: 15^a^ (superficial) Cohort: 9^a^ (superficial)	Baseline: 14.3% (superficial) Cohort: 8.7% (superficial)	Baseline: only for individual measures Cohort: only for individual measures
Frenette, 2016, Canada [42]	Cohort (before/after)	Hepatobiliary Surgery, Solid Organ Transplantation (liver, kidney, pancreas)	Baseline: 453 Cohort: 971	Baseline: 79 Cohort: 80	Baseline: 17.4% Cohort: 8.2%	Baseline: 45.1% (global) Cohort: 60.3% (global)
Garcell, 2017, Qatar^c^ [43]	Cohort	Appendix surgery	Baseline: 59 Cohort: 300	Baseline: NR Cohort: NR	Baseline: NR Cohort: NR	Baseline: only for individual measures Cohort: only for individual measures
Geubbels, 2004, Netherlands [44]	Cohort (before/after)	Appendix surgery	Baseline: NR Cohort: NR	Baseline: NR Cohort: NR	Baseline: 14.9% Cohort: 3.6%	Baseline: NR Cohort: 81% (individual measure)
Grant, 2018 (Epub 2017), US ^c^ [45]	Cohort (before/after)	Colorectal surgery	Baseline: 401 Cohort: 763	Baseline: NR Cohort: NR	Baseline: NR Cohort: NR	Baseline: NR Cohort: NR
Hechenbleikner, 2015, US [46]	Cohort (before/after)	Colorectal surgery	Baseline: Cohort: 387	Baseline: Cohort: 71	Baseline: 22.4% Cohort: 18.9%	Baseline: only for individual measures Cohort: only for individual measures
Hedrick, 2007, US [47]	Cohort (before/after)	Colorectal surgery	Baseline: 175 Cohort: 132	Baseline: 45 Cohort: 21	Baseline: 25.6% Cohort: 15.9%	Baseline: only for individual measures Cohort: only for individual measures
Hedrick, 2007, US [48]	Cohort (before/after)	Intra-Abdominal Surgery	Baseline: 379 Cohort: 390	Baseline: 35^a^ Cohort: 22^a^	Baseline: 9.2% Cohort: 5.6%	Baseline: only for individual measures Cohort: only for individual measures
Hewitt, 2017, US [49]	Cohort (before/after)	Colorectal surgery	Baseline: 489 Cohort: 212	Baseline: 68 Cohort: 10	Baseline: 13.9% Cohort: 4.7%	Baseline: NR Cohort: 80% (global)
Kao, 2010, US [50]	Cohort (controlled staggered before/after)	Colorectal surgery, abdominal hysterectomies, and abdominal vascular operations	Baseline: 91^a^ Cohort: 62^a^	Baseline: 4^a^ Cohort: 2^a^	Baseline: 4.4%^a^ (colorectal) Cohort: 3.2%^a^ (colorectal)	Baseline: NR (graphical only) Cohort: 63.7% (global)
Keenan, 2014, US [51]	Cohort (before/after)	Colorectal surgery	Baseline: 212 Cohort: 212	Baseline: 55^a^ Cohort: 18^a^	Baseline: 25.9%^a^ Cohort: 8.5%^a^	Baseline: NR Cohort: only for individual measures (narrative)
Keenan, 2015, US [52]	Cohort (before/after)	Colorectal surgery	Baseline: 337 Cohort: 285	Baseline: 116^a^ Cohort: 26^a^	Baseline: 34.4%^a^ Cohort: 9.1%^a^	Baseline: NR Cohort: only for individual measures
Kilan, 2017, Saudi Arabia [53]	Cohort (before/after)	Gastrointestinal surgery	Baseline: 55 Cohort: 214	Baseline: 5 Cohort: 11	Baseline: 9.1% Cohort: 5.1%	Baseline: 47.3% (global) Cohort: 82.2% (global)
Knox, 2016, Australia^c^ [54]	Cohort (before/after)	Abdominal general surgery	Baseline: 100 Cohort: 100	Baseline: NR Cohort: NR	Baseline: NR Cohort: NR	Baseline: 18% (global) Cohort: 15% (global)
Larochelle, 2011, US^c^ [55]	Cohort (before/after)	Colorectal surgery	Baseline: NR Cohort: 706	Baseline: NR Cohort: 87	Baseline: NR Cohort: 12.3%	Baseline: NR Cohort: only for individual measures
Lavu, 2012, US [56]	Cohort (before/after)	Pancreatic surgery	Baseline: 233 Cohort: 233	Baseline: 35 Cohort: 18	Baseline: 15.0% Cohort: 7.7%	Baseline: NR Cohort: narrative only (global)
Liau, 2010, Singapore [57]	Cohort (before/after)	Gastrointestinal and hernia surgery	Baseline: 1065^a^ Cohort: 2408	Baseline: 33^a^ Cohort: 12	Baseline: 3.1% Cohort: 0.5%	Baseline: NR Cohort: only for individual measures
Losh, 2017, US^c^ [58]	Cohort (before/after)	General, orthopedic, colorectal, oncological, OB/GYN, neurosurgery and urology surgical subspecialties	Baseline: NR Cohort: NR	Baseline: NR Cohort: NR	Baseline: 6.9%^b^ Cohort: 1.6%^b^	Baseline: NR Cohort: NR
Lutfiyya, 2012, US [59]	Cohort (before/after)	Colorectal surgery	Baseline: 430 Cohort: 195	Baseline: 91 Cohort: 13	Baseline: 21.16% Cohort: 6.67%	Baseline: NR Cohort: NR
Mammo, 2016, US^c^ [60]	Cohort (before/after)	Colorectal surgery	Baseline: 273 Cohort: 212	Baseline: 5^a^ (deep) Cohort: 0 (deep)	Baseline: 1.8% (deep) Cohort: 0% (deep)	Baseline: only for individual measures Cohort: only for individual measures
Misteli, 2012, Switzerland [61]	Cohort (before/after)	Vascular, visceral, and trauma surgery (Subgroup: Cholecystectomy and Colon Surgery)	Baseline: 483 Cohort: 257	Baseline: 44 Cohort: 25	Baseline: 9.1% Cohort: 9.7%^a^	Baseline: 41% (individual measure) Cohort: 56% (individual measure)
Nordin, 2018 (Epub 2017), US [62]	Cohort (before/after)	Pediatric gastrointestinal surgery	Baseline: NR Cohort: 1595	Baseline: NR Cohort: 75^a^	Baseline: 7.1% Cohort: 4.7%	Baseline: 43% (global) Cohort: 80% (global)
Pastor, 2010, US [63]	Cohort (early and late implementation period)	Colorectal Surgery	Baseline: 238 Cohort: 235	Baseline: 45 Cohort: 49	Baseline: 18.9% Cohort: 19.4%	Baseline: 30% (global) Cohort: 50% (global)
Pérez-Blanco, 2015, Spain [64]	Cohort (before/after)	Colorectal surgery	Baseline: 218 Cohort: 124	Baseline: 60 Cohort: 21	Baseline: 27.5% Cohort: 16.9%	Baseline: 62.6% (global) Cohort: 81,1% (global)
Reames, 2015, US ^c^ [65]	Cohort (before/after)	Abdominal general surgery	Baseline:14,005 Cohort: 14,801	Baseline: 449^a^ (superficial) Cohort: 474^a^ (superficial)	Baseline: 3.2% (superficial) Cohort: 3.2% (superficial)	Baseline: NR Cohort: NR
Tanner, 2016, UK [66]	Cohort (before/after)	Colorectal surgery	Baseline: 127 Cohort: 166	Baseline: 31 Cohort: 46	Baseline: 24% Cohort: 28%	Baseline: only for individual measures Cohort: 19% (global)
Tillman, 2013, US [67]	Cohort (before/after)	Cardiac surgery, colorectal surgery, general surgery (non-colorectal non-vascular), gynecologic surgery, orthopedic surgery, thoracic surgery, and vascular surgery	Baseline: 79 Cohort: 104	Baseline: 19 Cohort: 12	Baseline: 24.1% (colorectal) Cohort: 11.5% (colorectal)	Baseline: only for individual measures Cohort: only for individual measures
Vogel, 2010, Germany [68]	Cohort (before/after)	Colorectal surgery	Baseline: 332 Cohort: 341	Baseline: 26 Cohort: 12	Baseline: 7.8% Cohort: 3.5%	Baseline: NR Cohort: narrative only (global)
Vu, 2018 (Epub 2017), US [69]	Cohort (before/after)	Colorectal surgery	Baseline: NR Cohort: NR	Baseline: NR Cohort: NR	Baseline: 6.7% Cohort: 3.9%	Baseline: only for individual measures Cohort: only for individual measures
Waters, 2017, US [70]	Cohort (before/after)	Colorectal surgery	Baseline: 2408 Cohort: 2873	Baseline: 193^a^ Cohort: 172^a^	Baseline: 8% Cohort: 6%	Baseline: only for individual measures Cohort: only for individual measures
Wick, 2012, US [71]	Cohort (before/after)	Colorectal surgery	Baseline: 278 Cohort: 324	Baseline: 76 Cohort: 59	Baseline: 27.3% Cohort: 18.2%	Baseline: only for individual measures Cohort: only for individual measures
Wick, 2015, US [72]	Cohort (before/after)	Colorectal surgery	Baseline: 310 Cohort: 330	Baseline: 59^a^ Cohort: 24^a^	Baseline: 18.8% Cohort: 7.3%	Baseline: NR Cohort: NR
Willis, 2016, US [73]	Cohort (before/after)	Appendix surgery (Pediatric Complicated Appendicitis)	Baseline: 191 Cohort: 122	Baseline: 50^a^ Cohort: 14^a^	Baseline: 26.2%^a^ Cohort: 11.4%^a^	Baseline: NR Cohort: 87.5% (global)

*Notes:* NR Not reported; ^a^ self-calculated; ^b^ all cases (abdominal cases were not separately reported); ^c^ not included in effectiveness analysis due to missing overall baseline or cohort. SSI rates or not separately reported abdominal SSI rates

Thirty-two studies provided at least some information on baseline and/or cohort compliance [34–36, 38, 41–44, 46–57, 60–64, 66–71, 73]. However, these reports varied considerably regarding quality, scope, and time frame. Twenty-three studies addressed baseline and cohort compliance, while nine addressed cohort compliance [44, 49, 51, 52, 55–57, 68, 73]. In some of these studies, the compliance rates were reported in narrative or graphical form only, which made it difficult to determine the exact rates. In addition, from those studies that reported explicit baseline and cohort compliance rates, 13 studies reported rates for individual measures, while 7 presented global rates for all clinical measures [35, 38, 42, 53, 54, 62, 64]; only two studies reported both types of rates [34, 63]. Thus, further analyses on the associations between implementation interventions and compliance with clinical interventions are omitted.

In contrast, 35 studies reported baseline and cohort SSI rates [34, 36–42, 44, 46–53, 56–73]. Three studies only reported specific rates, such as superficial or deep SSI rates [41, 60, 65], while in 27 of the 35 studies, the overall SSI rates were explicitly reported. In five studies, it was necessary to calculate the overall rates [50–52, 61, 73]. One of the four studies that considered surgical specialties in addition to abdominal surgery did not report SSI rates specific for abdominal surgery [58]. Eventually, the overall baseline and cohort SSI rates from 31 studies could be determined. Furthermore, SSI reporting periods varied; thus, the SSI rates were calculated for varying time frames. For instance, some studies reported pre-intervention and post-intervention SSI rates, whereas others reported SSI rates for the total study period or several periods. Due to these differences, the earliest available SSI rate was considered to represent the baseline rate, and the last available rate was considered the cohort rate. Additionally, while some studies reported SSI rates adjusted for confounders, others did not, and still others did not clarify whether the reported rates were adjusted or not. For this reason, rates from the model with the highest number of confounders were used in the present analysis. Finally, as noted, the “Quality Assessment Tool for Before-After (Pre-Post) Studies With No Control Group” [31] was used. With a quality score of 12 as the highest possible score, the mean score was 7, with a 95% CI of 6.7–7.3, a standard deviation of 0.96 and a range between 4 and 9 (see Additional file 3).

### Distribution of implementation interventions

Table 3 presents the classifications of the implementation interventions identified in the included studies into the categories specified in the EPOC Taxonomy. Both the quantity and types of implementation interventions varied strongly across the studies. The five most frequently used implementation interventions were audit and feedback (80%, 32/40), organizational culture (70%, 28/40), monitoring the performance of the delivery of healthcare (65%, 26/40), reminders (52.5%, 21/40) and educational meetings (45%, 18/40). The categories not coded at all were the following: clinical incident reporting, educational games, patient-mediated interventions, public release of performance data, and routine patient-reported outcome measures. The number of implementation interventions ranged from one to ten (see Table 3), with a mean of 4.6 (median: 4, mode: 2) and a standard deviation of 2.5. For three studies, only one EPOC category was coded. According to the aforementioned definition (at least three and usually five components [22]), 29 studies employed a multimodal strategy (with 13 studies using six or more strategies). Among these, ten studies used a combination that included the five top-coded implementation interventions. The quality of implementation intervention reporting varied strongly across studies. Some studies reported the implementation interventions in great detail, whereas others merely named them.

**Table 3:** EPOC implementation strategies [26] identified in the *N* = 40 included studies

First author, year	Audit and feedback	Organi-sational culture	Monito-ring the perfor-mance of the delivery of healthcare	Re-minders	Educa-tional meetings	Local con-sensus processes	Continous quality improve-ment	Local opinion leaders	Edu-cational materials	Clinical practice guidelines	Educa-tional outreach visits, or academic detailing	Commu-nities of practice	Tailored inter-ventions	Mana-gerial super-vision	Interpro-fessional education	Clinical incident reporting	Educa-tional games	Patient-mediated inter-ventions	Public release of performance data	Routine patient-reported outcome measures	Number of imple-mentation strategies used in studies
Bull, 2011 [34]	X	X	X	X	X	X	X	X													8
Cameron, 2015 [35]	X								X												2
Cima, 2013 [36]	X	X	X	X	X	X	X														7
Connolly, 2016 [37]		X		X	X	X															4
Crolla, 2012 [38]	X	X	X											X							4
DeHaas, 2016 [39]		X									X										2
Elia-Guedea, 2017 [40]	X	X		X	X																4
Forbes, 2008 [41]	X	X	X			X		X													5
Frenette, 2016 [42]	X		X	X		X				X											5
Garcell, 2017 [43]	X		X	X	X																4
Geubbels, 2004 [44]	X		X																		2
Grant, 2018 (Epub 2017) [45]		X																			1
Hechenbleikner, 2015 [46]	X	X			X																3
Hedrick, 2007 [47]	X	X	X	X	X	X															6
Hedrick, 2007 [48]	X	X	X	X	X	X		X													7
Hewitt, 2017 [49]		X			X	X															3
Kao, 2010 [50]	X	X		X	X								X								5
Keenan, 2014 [51]	X		X				X														3
Keenan, 2015 [52]	X	X	X	X				X													5
Kilan, 2017 [53]	X	X	X	X	X	X							X		X						8
Knox, 2016 [54]				X					X												2
Larochelle, 2011 [55]	X	X	X																		3
Lavu, 2012 [56]		X																			1
Liau, 2010 [57]	X	X	X	X	X	X			X	X											8
Losh, 2017 [58]	X	X	X	X	X		X	X				X									8
Lutfiyya, 2012 [59]	X	X	X	X	X		X														6
Mammo, 2016 [60]					X					X											2
Misteli, 2012 [61]				X		X															2
Nordin, 2018 (Epub 2017) [62]	X		X																		2
Pastor, 2010 [63]	X	X	X	X			X				X		X								7
Pérez-Blanco, 2015 [64]	X																				1
Reames, 2015 [65]	X	X	X	X	X		X	X	X			X		X							10
Tanner, 2016 [66]	X		X					X													3
Tillman, 2013 [67]	X	X	X		X				X												5
Vogel, 2010 [68]	X	X	X	X			X														5
Vu, 2018 (Epub 2017) [69]	X	X	X			X	X	X			X	X									8
Waters, 2017 [70]	X		X																		2
Wick, 2012 [71]	X	X	X	X	X		X	X	X												8
Wick, 2015 [72]	X	X	X	X		X	X	X			X										8
Willis, 2016 [73]	X	X				X				X											4
**Number of studies the implementation strategy was used in**	32	28	26	21	18	14	11	10	6	4	4	3	3	2	1	0	0	0	0	0	
**% of studies the implementation strategy was used in**	80%	70%	65%	52.5%	45%	35%	27.5%	25%	15%	10%	10%	7.5%	7.5%	5%	2.5%	0%	0%	0%	0%	0%	

### Effectiveness of implementation interventions

A challenge for the evaluation of the effectiveness of implementation interventions was the differences in outcome reporting across studies. In particular, the compliance rates were incompletely reported. In 8 of 9 studies that explicitly reported global baseline and cohort compliance rates, the global compliance rates increased [34, 35, 38, 42, 53, 62–64]. Increases ranged from 15.2 to 70% (mean value: 31.7%). In another study, the global compliance rate decreased by 3% [54].

Due to these difficulties, an effectiveness analysis focused on SSI rates. In 28 of the 31 studies that reported overall baseline and cohort SSI rates, there was a decrease in SSI rates from baseline to cohort [34, 36–40, 42, 44, 46–53, 56, 57, 59, 62, 64, 67–73], with absolute SSI risk differences ranging from − 1.2% to − 25.3%. Three studies [61, 63, 66] reported an increase in SSI rates from baseline to cohort (range: + 0.5% to + 4%). The reasons for these increases were not always clear. In one study, the implementation interventions failed to improve compliance with clinical measures, and the authors suspected that the implementation phase was too short [66]. In the second study, the SSI rate increased even though bundle compliance increased. The authors noted that their sample size was small and that the antibiotic prophylaxis selection was changed in the cohort period [63]. In the third study, in which the SSI rate increased slightly even though implementation interventions increased compliance, the authors indicated that their sample size was possibly too small [61].

**Fig. 3:**
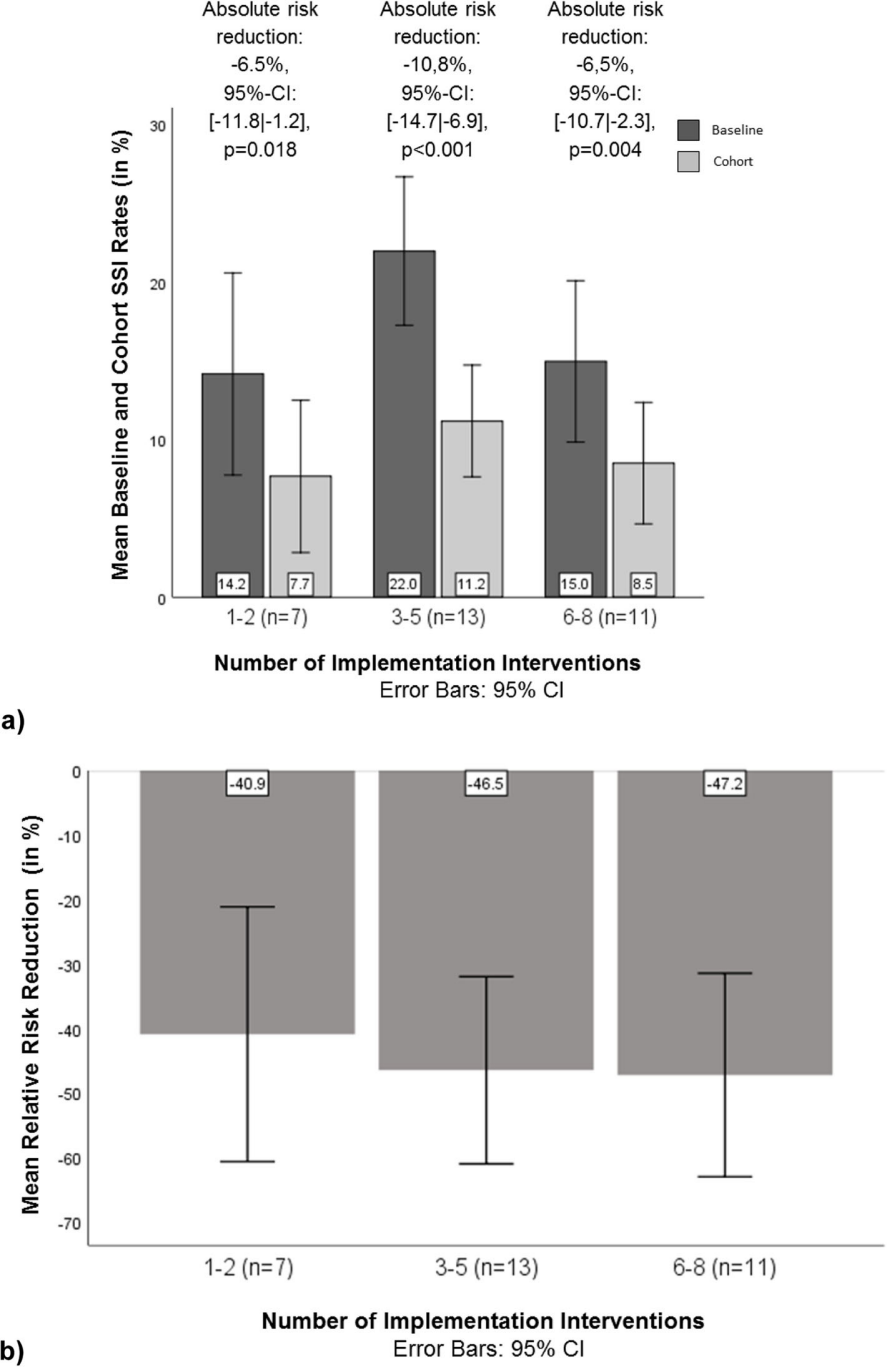
**a**) Mean baseline and cohort SSI rates and absolute risk reductions for *N* = 31 studies* with different numbers of implementation interventions; **b**) Mean relative risk reductions for *N* = 31 studies* with different numbers of implementation interventions. *Note*: * only studies reporting both baseline and cohort SSI rates

Thirty-one studies that reported overall SSI rates for abdominal surgery were included in the quantitative analysis. Pertaining to the research question on potential differences in the SSI-preventive effectiveness of studies with different implementation intervention bundle sizes, Fig. 3a shows that there was a decrease in SSI rates from baseline to cohort in every group defined by the number of EPOC categories coded. This analysis showed an absolute SSI risk reduction of 6.5% for the group with 1–2 types of implementation interventions, a reduction of 10.8% for those with 3–5 types, and a reduction of 6.5% for the group with 6–8 types. All reductions reached statistical significance. While the largest absolute SSI risk difference pertained to the group with 3–5 implementation interventions, the reductions did not significantly differ from one another across the three groups (*p* = 0.236). Finally, while no two groups of studies differed significantly in cohort rates, the baseline rates for studies with 3–5 interventions differed significantly from those with 6–8 interventions (*p* = 0.049), while the contrast between 3 and 5 types and 1–2 types failed to reach statistical significance (*p* = 0.055; see Table 4). The analysis of relative SSI risk reductions showed a relative reduction of 40.9% for the group with 1–2 implementation interventions, 46.5% for the group with 3–5 interventions, and 47.2% for the group with 6–8 elements (see Fig. 3b). These reductions did not significantly differ from one another across the three groups (*p* = 0.862).

**Table 4:** Tests for significance of differences in baseline and cohort SSI rates between *N* = 31 studies^a^ with different numbers of implementation interventions

	Studies with 3–5 vs. 1–2 implementation interventions			Studies with 6–8 vs. 1–2 implementation interventions			Studies with 3–5 vs. 6–8 implementation interventions		
	Difference	95%-confidence interval	*p*-value	Difference	95%- confidence interval	*p*-value	Difference	95%- confidence interval	*p*-value
baseline	7.8%	[−0.2│15.8]	*p* = 0.055	0.8%	[−7.4│9.0]	*p* = 0.843	7.0%	[0.1│14.0]	*p* = 0.049
cohort	3.5%	[−2.5│9.5]	*p* = 0.241	0.8%	[−5.4│7.0]	*p* = 0.785	2.7%	[−2.6│7.9]	*p* = 0.305

*Note*: ^a^ only studies reporting both baseline and cohort SSI rates

**Fig. 4:**
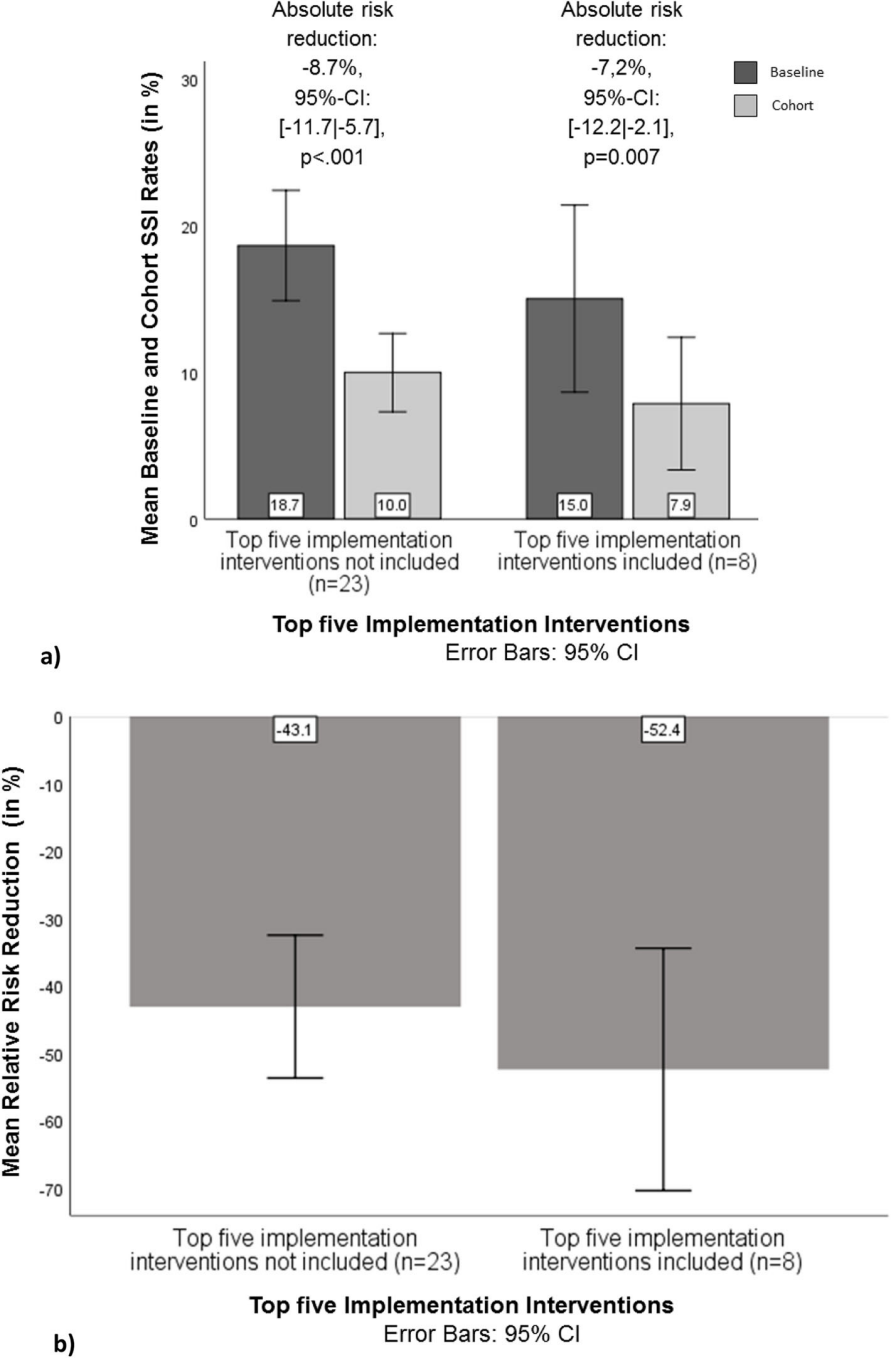
**a**) Mean baseline and cohort SSI rates and absolute risk reductions for *N* = 31 studies* with or without all five implementation interventions most used across the studies (“top five”); **b**) Mean relative risk reductions for *N* = 31 studies* with or without all five implementation interventions used most across studies (“top five”). *Note*: * only studies reporting both baseline and cohort SSI rates

Pertaining to possible differences in SSI-preventive effectiveness between studies including implementation interventions that were most frequently used across all studies (audit and feedback, organizational culture, monitoring the performance of the delivery of healthcare, reminders, educational meetings) vs. those that were not, Fig. 4a shows there was a decrease in SSI rates from baseline to cohort in the groups with and without the top five interventions, with an absolute SSI risk reduction of 7.2% in the former and of 8.7% in the latter group (*p* < 0.01 in both cases). Neither the baseline and cohort rates (see Table 5) nor the relative risk reductions (*p* = 0.602) significantly differed from one another across the groups. Regarding relative SSI risk reductions, the analyses showed a reduction of 52.4% for studies that used all five of the most frequently coded implementation interventions and a reduction of 43.1% for those who did not (see Fig. 4b). While these differences did not significantly differ from one another across the groups (*p* = 0.369), the former was the only relative risk reduction that significantly differed from − 33.3%, i.e., the 25th percentile of the overall relative risk reduction distribution (*p* = 0.017). Also not shown here are the results contrasting the studies with the top three or four implementation interventions, vs. those not including these interventions, which showed smaller risk reductions but patterns comparable to those seen in the top five-analysis.

**Table 5:** Tests for significance of differences in baseline and cohort SSI rates between *N* = 31 studies^a^ with or without top five implementation interventions

	Studies with top five implementation interventions vs. studies without		
	Difference	95%-confidence interval	*p*-value
baseline	−3.6%	[−11.0│3.8]	*p* = 0.324
cohort	−2.1%	[−7.4│3.1]	*p* = 0.418

*Note*: ^a^ only studies reporting both baseline and cohort SSI rates

## Discussion

This systematic review aimed to identify implementation interventions that are employed in the field of abdominal surgery to implement measures to prevent SSIs. On average, studies used almost five implementation interventions, and nearly half of the studies reported five or more. The most frequently used interventions were audit and feedback, measures related to organizational culture (e.g., multidisciplinary teams), monitoring, reminders, and educational meetings. An effectiveness analysis revealed significant absolute and relative SSI-risk reductions but did not reveal significant differences in risk reduction either by the number of implementation interventions used or by use of the five most frequent ones vs. not. Descriptively, the largest absolute risk reduction difference pertained to studies with three to five implementation interventions; however, these studies started from a higher baseline rate than did studies employing either less or more interventions. In contrast, descriptively the largest relative SSI risk reduction was found in the group with at least six implementation interventions. Regarding studies using all five of the most used implementation interventions, descriptively higher absolute risk reductions pertained to the group that did not use them, while the relative reduction was highest in the group of studies that used them.

The results regarding the distribution of implementation interventions applied are mostly consistent with findings from other reviews. Most prominently, the review by Ariyo et al. on implementation strategies in SSI prevention also generally found multidisciplinary work, educational approaches, and monitoring and feedback among the foremost interventions in the 125 studies they included [23]. Additionally, Borgert and colleagues, who examined intensive care bundles [28], and Wuchner, who examined translating evidence into nursing practices [74], found educational measures, reminders, and audit and feedback to be most frequently used. On one hand, combined with the finding that only 27.5% of studies included in the present review did not use multimodal strategies, these results indicate that the latter implementation approach has reached the abdominal surgery field, at least in terms of published studies. On the other hand, the specific types of interventions found to be frequently used indicate that most strategies represent what Srigley and colleagues for hand hygiene have termed “standard multimodal programmes” [75], i.e., those that rely heavily on education and reminders (in addition to hand hygiene product availability). These standard measures may be chosen as “off-the-shelf” options that are not selected based on any specific theory or previous analysis but merely because they have been applied before or are judged as feasible, as The Improved Clinical Effectiveness through Behavioural Research Group (ICEBeRG) has described this risk [76].

Among the other interventions least used (i.e., in below 10% of the studies), tailored interventions, which have, e.g., been shown to be especially effective in promoting hand hygiene compliance and lead to larger reductions in MRE-infections than non-tailored ones [77, 78], are particularly noteworthy. Baker and colleagues defined tailored interventions as “… strategies to improve professional practice that are planned, taking account of prospectively identified determinants of practice” [79] (p. 5). While none of the three studies coded to have used tailoring addressed the concept explicitly [50, 53, 63], they implemented their respective implementation interventions based on previously identified barriers and thus were regarded to have used this strategy. The multitude of clinical interventions regarding the prevention of SSI has thus far impeded the use of tailoring. In this context, further studies should elucidate ways to tailor SSI-preventive interventions (e.g., the “Surgical Site Infections and Antibiotics Consumption in Surgery”-[“WACH”-]trial [80]).

The effectiveness analysis reported both absolute and relative risk reductions, thus taking into account risk communication research showing that the presentations of the two types of risk reduction tend to have differential effects, notably to improve understanding (absolute reductions) and to promote acceptance of interventions (relative reductions) [81]. In this context, disparities in the results of the effectiveness analyses in relation to absolute vs. relative risk reductions found in this review should not be over-interpreted. However, it may still be noteworthy that descriptively, absolute risk reduction was highest in the studies with three to five interventions, while the relative risk reduction was highest among those studies with at least six interventions; simultaneously, studies with all five most frequently used interventions had a lower absolute but higher relative risk reduction than those that did not include these interventions. As mentioned before, the WHO recommends the use of multimodal strategies with three or more (usually five) different types of implementation interventions for implementing infection prevention and control activities in acute health care facilities [21, 22]. However, for clinical interventions in colorectal surgery [14, 82] the identification of specific combinations of interventions, let alone any “the more, the better”, e.g., linear dose-response-association, remains difficult. However, SSI prevention initiatives may take away from the present results that following the recommendation to include three to five implementation interventions, and/or audit and feedback, organizational culture, monitoring, reminders, and educational meetings (as the most “standard” interventions), most likely does not represent preventive malpractice.

Finally, implementation interventions coded into one and the same EPOC category—e.g., audit and feedback, organizational culture, and reminders—varied “phenotypically”. In the case of audit and feedback, for instance, feedback was provided through newsletters [38] or through postings of performance figures [41] or SSI rates and compliance data [66] in areas such as operating rooms. In contrast, in other studies, feedback was provided in clinical meetings [34] or feedback sessions [42]. At the same time, not only the tools but also the feedback frequency varied; in most cases, it was provided monthly [e.g., 36, 41, 43, 47, 53, 66, 71, 73], whereas in others, it was provided quarterly [38, 55], semi-annually [44, 70], or annually [42] (see Additional file 4). Furthermore, in some studies, individual SSI and/or compliance data were provided to the staff, while in other studies, overall data or both individual and overall data were made available. At the same time, while measures that aimed to trigger changes in organizational culture varied as well, they mostly focused on building groups such as multidisciplinary teams [36, 39, 48, 53, 55, 72], multidisciplinary task forces [63], steering committees [34], and dedicated operating room teams [45]. For instance, teams were formed to develop bundles of clinical measures, promote implementation, or monitor compliance. Other tools of organizational culture were introducing preoperative and postoperative briefings [56], extended timeouts [50], and promoting a safety culture by including feedback in terms of correcting each other [38]. Finally, reminders varied in that sometimes checklists were used [50, 68], while in other studies, standardized tables for antibiotic prophylactic or prophylactic guidelines were posted or displayed in operating rooms [54, 59] or automatic electronic reminders reminded staff about antibiotic re-dosing [36, 61, 63]. Some studies used more uncommon tools, such as dressing stickers with change instructions [37] or personal reminders given noncompliance [53]. Altogether, while the EPOC implementation strategies taxonomy allows for a more differentiated description of SSI-preventive studies and programmes than does the “four Es” approach, an even more fine-grained system of implementation interventions may be of added value, e.g., the taxonomy for behaviour change techniques proposed by Michie and colleagues [83].

### Limitations

Several limitations have to be noted. First, due to the heterogeneities across the studies both in the number and types of implementation interventions reported, the clinical interventions examined, outcome reports, and methodical quality in terms of risk of bias, meta-analysis was not feasible. Additionally, identification of the most effective bundle of implementation interventions or the most effective single implementation intervention was not possible. Notably, for implementation interventions used in almost all or no studies, there is no sufficiently large comparison group. Moreover, no implementation intervention was particularly frequent in studies with three to five implementation interventions, but for each intervention, the probability of being included in a study increased with the implementation bundle size. Related to this issue, it was difficult to determine whether outcomes were related to differences in implementation or clinical interventions or both. Eldh and colleagues have called attention to this problem by describing the conceptual “greyness” between clinical and implementation interventions that arises, e.g., from differences in study design, study performance, or reporting processes [84]. For example, if a reminder is employed to promote compliance with antibiotic prophylaxis and at the same time antibiotic agents are changed, it may remain unclear whether outcomes are attributable to the implementation intervention, the clinical intervention, or both. Other reviewers have had similar problems with such heterogeneities when evaluating the effectiveness of implementation interventions [28, 74, 85]. In this context, Borgert et al. have recommended that quality improvement studies should be reported in a unified way to be able to compare results [28], a call which the results and limitations of the present review emphasize. In other words, a reporting priority should be the exact description of implementation interventions and strategies. To this end, Proctor et al. have proposed a framework for reporting implementation strategies in which they recommend labelling, defining and specifying interventions and strategies by specifying actors, actions, action targets (e.g., target groups), temporality, dose, outcomes, and justification of interventions [86]. This framework is also recommended for the relevant item in the Standards for Reporting Implementation Studies (StaRi) checklist (item 9 “description of implementation strategies”) [87, 88]. To highlight the richness of details (or lack thereof) and discrepancies across the studies included in this review, we used the relevant parts of this framework to describe, by way of example, the reporting quality of the implementation intervention most frequently coded, i.e., audit and feedback. Specifically, studies were screened regarding the reporting of actors, actions, target groups, temporality, and dose. As Additional file 4 shows, the audit and feedback category was described rather differently in the studies, and large gaps pertain to the reporting of actors (e.g., who is given feedback?), target groups (e.g., who is getting feedback?), and temporality (e.g., when was feedback given?). Thus, if the studies had reported the interventions in higher accordance with the framework, it would have been possible to determine key information, e.g., to perform a more comprehensive effectiveness analysis.

Second, the present review could not be limited to studies that had explicitly focused on the effectiveness of implementation interventions. This was because a preliminary search showed that in abdominal surgery, only very few studies were performed with the primary aim of examining the effectiveness of implementation interventions to promote compliance with clinical interventions to prevent SSIs. While the inclusion of studies not focusing on implementation interventions broadened the scope of this review, it had the effect that implementation interventions were often poorly reported (see above). That is, at times, it was difficult to identify the implementation interventions because they had not been designated as such or reported in an unstructured manner in more than one part of the publication. In addition, it can be assumed that some studies used implementation interventions but did not report them because they were omitted during the review process. Additionally, the possibility of publication bias has to be taken into account since only three studies reported an increase in SSI rates from baseline to cohort after intervention [61, 63, 66]. All told, these considerations call for more implementation research in SSI prevention both generally and in abdominal surgery in particular.

Finally, it must be re-iterated that only the implementation strategies part of the EPOC Taxonomy was used to assess the included studies. While this restriction was deliberate due to this review’s focus on implementation interventions, further analysis should describe SSI-preventive studies and programmes in regard to the other three superordinate categories of the EPOC Taxonomy, i.e., delivery arrangement (e.g., how and when care is delivered), financial arrangements (e.g., collection of funds, the purchase of services, and the use of targeted financial incentives or disincentives), and governance arrangements (e.g., rules or processes that affect the way in which powers are exercised) [26].

## Conclusion

In abdominal surgery, mostly multimodal strategies with standard implementation interventions representing audit and feedback, organizational culture, performance monitoring, reminders, and educational meetings are used. At the same time, some implementation interventions such as tailoring were seldom or not at all used, thus indicating potential leeway for further gains in SSI prevention by overcoming standard multimodal strategies. The effectiveness analysis regarding bundle size and composition (in terms of including the five most used interventions) did not render definite results. However, descriptively the highest absolute risk reductions were found for studies with three to five interventions used and the highest relative risk reduction in studies with all top five interventions. Further studies are needed to determine the types and quantities of implementation interventions that are especially effective in promoting compliance with measures to prevent SSIs in abdominal surgery. In this context, it is advisable for future studies to report implementation interventions in a more standardized fashion, e.g., in a separate paper section using the EPOC Taxonomy [26] and the framework from Proctor et al. [86], even in cases when the primary focus of the report is different.

## Supplementary information

**Additional file 1:Table S1.** Search Strategy

**Additional file 2: Table S2.** Clinical interventions (SSI preventive measures) in the *N* = 40 included studies

**Additional file 3:Table S3.** Quality assessment using the Quality Assessment Tool for Before-After (Pre-Post) Studies With No Control Group by the National Heart, Lung, and Blood Institute [31]

**Additional file 4:Table S4.** Reporting of the implementation intervention audit and feedback

## Data Availability

The datasets used and/or analysed during the current study are available from the corresponding author on reasonable request.

## Abbreviations

ECDC: European Centre for Disease Prevention and Control
EPOC: Effective Practice and Organisation of Care
GLM: General Linear Model
ICEBeRG: Improved Clinical Effectiveness through Behavioural Research Group
PRISMA: Preferred Reporting Items for Systematic Reviews and Meta-Analysis
SSI: Surgical Site Infection
StaRi: Standards for Reporting Implementation Studies
UK: United Kingdom
US: United States
WACH: “Surgical Site Infections and Antibiotics Consumption in Surgery”-trial (German title: Wundinfektionen und Antibiotikaverbrauch in der Chirurgie)
WHO: World Health Organization
