# Supplementary material for: Implementation interventions in preventing surgical site infections in abdominal surgery: a systematic review
Source: BMC Health Serv Res. 2020 Mar 20;20:236. doi: 10.1186/s12913-020-4995-z (PMC7083020; doi:10.1186/s12913-020-4995-z)
Supplement: Supplementary file 1 — Additional file 1:Table S1. Search Strategy [file 12913_2020_4995_MOESM1_ESM.pdf]

Additional file 1

Table S1: Search Strategy

PubMed

Topic	Search terms and connection
**Abdominal surgery**	("abdominal surgery" OR "abdominal operation" OR "abdominal procedure" OR "visceral surgery" OR "digestive system surgical procedures" [Mesh] OR "digestive system" [Mesh] OR abdom* OR gastrointestin* OR colorect* OR colect* OR esophagect* OR gastrect* OR pancreatect* OR pancreaticoduodenect* OR hepatect* OR appendect*)
	**AND**
**Surgical site infections**	("surgical wound infection" [Mesh] OR "surgical site infection" OR "surgical site infections" OR SSI OR SSIs OR "postoperative wound infection")
	**AND**
**Guideline implementation /compliance**	("Guideline Adherence" [Mesh] OR guideline OR guideline* OR "practice guideline" OR "clinical guideline" OR recommend* OR compliance OR adherence OR adopt* OR implemen* OR improv* OR dissemin* OR “implementation intervention" OR "implementation tool" OR "implementation strategy" OR "implementation program" OR "implementation research" OR "implementation science" OR "knowledge translation" OR "knowledge circulation" OR bundle OR bundles OR bundl* OR "Patient Care Bundles" [Mesh])
	**AND**
**Implementation interventions**	(intervent* OR motivat* OR "behavior change" OR educat* OR promot* OR leaders OR audit OR feedback OR remind* OR marketing OR "mass media" OR trainings OR meetings OR sessions OR workshops OR posters OR handouts OR checklists OR "decision support systems" OR "Internet based learning" OR "multidisciplinary teams" OR "skill mix change" OR "economic incentives" OR "organizational change" OR "organizational culture" OR "tailored interventions" OR "behavioral change" OR leader OR audits OR feedbacks OR training OR meeting OR session OR workshop OR poster OR handout OR checklist OR "decision support system" OR "multidisciplinary team" OR "skill mix changes" OR "economic incentive" OR "tailored intervention" OR incentive OR tailor* OR "behavioral pattern" OR remind OR communicat* OR measure OR measures OR bundle OR bundles OR bundl* OR "Patient Care Bundles" [Mesh])

Web of Science Core Collection

Topic	Search terms and connection
**Abdominal surgery**	("abdominal surgery" OR "abdominal operation" OR "abdominal procedure" OR "visceral surgery" OR "digestive system surgical procedures" OR "digestive system" OR abdom* OR gastrointestin* OR colorect* OR colect* OR esophagect* OR gastrect* OR pancreatect* OR pancreaticoduodenect* OR hepatect* OR appendect*)
	**AND**
**Surgical site infections**	("surgical wound infection" OR "surgical site infection" OR "surgical site infections" OR SSI OR SSIs OR "postoperative wound infection")
	**AND**
**Guideline implementation /compliance**	("Guideline Adherence" OR guideline OR guideline* OR "practice guideline" OR "clinical guideline" OR recommend* OR compliance OR adherence OR adopt* OR implemen* OR improv* OR dissemin* OR “implementation intervention" OR "implementation tool" OR "implementation strategy" OR "implementation program" OR "implementation research" OR "implementation science" OR "knowledge translation" OR "knowledge circulation" OR bundle OR bundles OR bundl* OR "Patient Care Bundles")
	**AND**
**Implementation interventions**	(intervent* OR motivat* OR "behavior change" OR educat* OR promot* OR leaders OR audit OR feedback OR remind* OR marketing OR "mass media" OR trainings OR meetings OR sessions OR workshops OR posters OR handouts OR checklists OR "decision support systems" OR "Internet based learning" OR "multidisciplinary teams" OR "skill mix change" OR "economic incentives" OR "organizational change" OR "organizational culture" OR "tailored interventions" OR "behavioral change" OR leader OR audits OR feedbacks OR training OR meeting OR session OR workshop OR poster OR handout OR checklist OR "decision support system" OR "multidisciplinary team" OR "skill mix changes" OR "economic incentive" OR "tailored intervention" OR incentive OR tailor* OR "behavioral pattern" OR remind OR communicat* OR measure OR measures OR bundle OR bundles OR bundl* OR "Patient Care Bundles")
