# Supplementary material for: Implementation interventions in preventing surgical site infections in abdominal surgery: a systematic review
Source: BMC Health Serv Res. 2020 Mar 20;20:236. doi: 10.1186/s12913-020-4995-z (PMC7083020; doi:10.1186/s12913-020-4995-z)
Supplement: Supplementary file 2 — Additional file 2: Table S2. Clinical interventions (SSI preventive measures) in the N = 40 included studies [file 12913_2020_4995_MOESM2_ESM.pdf]

Additional file 2

Table S2: Clinical interventions (SSI preventive measures) in the N=40 included studies

First author, year	Clinical interventions (SSI preventive measures)
Bull, 2011 [34]	- Normothermia<br>- Oxygen delivery<br>- Blood pressure monitoring<br>- Diabetic control<br>- Antibiotic prophylaxis
Cameron, 2015 [35]	- Antibiotic prophylaxis
Cima, 2013 [36]	- Patient cleansing<br>- Antibiotic prophylaxis<br>- Chloraprep (incisional area)<br>- Closing tray<br>- Glove change before closure<br>- Hand hygiene<br>- Dressing removal
Connolly, 2016 [37]	- Bowel preparation<br>- Shower with chlorhexidine<br>- Chlorhexidine antimicrobial scrub of abdomen<br>- Glucose control<br>- Hair removal<br>- Surgical masks<br>- Minimize traffic and the OR door “left open” time<br>- Chlorhexidine skin prep, extended skin prep area<br>- Hand hygiene<br>- Clean scrubs<br>- Antibiotic prophylaxis<br>- Normothermia<br>- Incision drape<br>- Wound retractor<br>- Before closing: re-glove, re-prep, re-towel incision area, use clean instrument tray<br>- Wound closure technique<br>- Dressing changes
Crolla, 2012 [38]	- Antibiotic prophylaxis<br>- Hair removal<br>- Normothermia<br>- Discipline in the operating room
DeHaas, 2016 [39]	- Antibiotic prophylaxis<br>- Preoperative warming<br>- Hair removal<br>- Chlorhexidine showers and wipes<br>- Mechanical bowel preps<br>- Normothermia<br>- Chlorhexidine-alcohol surgical prep<br>- Wound protectors<br>- Clean closure trays<br>- Gown and/or glove changes as appropriate<br>- Redosing of antibiotics at appropriate time intervals<br>- Dressing removal
Elia-Guedea, 2017 [40]	- Antibiotic prophylaxis<br>- Restriction of staff transit in the operating room<br>- Handling of wounds (dressings, gloves, surgical tools)<br>- Wound care
Forbes, 2008 [41]	- Antibiotic prophylaxis<br>- Normothermia<br>- Glucose control
Frenette, 2016 [42]	- Antibiotic prophylaxis<br>- Hair removal<br>- Shower with chlorhexidine<br>- Skin antisepsis<br>- Operating room ventilation<br>- Hand washing<br>- Sterile clothing<br>- Sterile operating room and equipment
Garcell, 2017 [43]	- Antibiotic prophylaxis<br>- Antimicrobial consumption
Geubbels, 2004 [44]	- Antibiotic prophylaxis (Hospital E, abdominal surgery)
Grant, 2018 (Epub 2017) [45]	- Preoperative, intraoperative and postoperative process measures (Enhanced Recovery after Surgery for colorectal surgery program)
Hechenbleikner, 2015 [46]	- Antibiotic prophylaxis<br>- Normothermia<br>- Chlorhexidine washcloth<br>- Skin preparation<br>- Mechanical bowel preparation

First author, year	Clinical interventions (SSI preventive measures)
Hedrick, 2007 [47]	- Antibiotic prophylaxis<br>- Normothermia<br>- Glucose control
Hedrick, 2007 [48]	- Antibiotic prophylaxis<br>- Normothermia<br>- Glucose control
Hewitt, 2017 [49]	- Smoking cessation<br>- Antibiotic prophylaxis<br>- Chlorhexidine gluconate cloths (night before surgery)<br>- Chlorhexidine gluconate cloths (day of surgery)<br>- Normothermia<br>- Hair removal<br>- Skin preparation with Chlorhexidine<br>- Glucose control<br>- Oxygen delivery<br>- Closing tray<br>- Changing gowns, and gloves before fascia closure<br>- Dressing down within 48 hours<br>- Consultation for all ostomies and complex wounds
Kao, 2010 [50]	- Antibiotic prophylaxis
Keenan, 2014 [51]	- Chlorhexidine shower<br>- Mechanical bowel preparation<br>- Antibiotic prophylaxis<br>- Preparation with chlorhexidine alcohol<br>- Fascial wound protector<br>- Dedicated wound closure tray<br>- Limited OR traffic<br>- Gown and glove change before fascial closure<br>- Glucose control<br>- Normothermia<br>- Removal of sterile dressing within 48 h<br>- Daily washings of incisions with chlorhexidine (postoperative)<br>- Patient education
Keenan, 2015 [52]	- Mechanical bowel preparation<br>- Chlorhexidine wipes<br>- Antibiotic prophylaxis<br>- Surgical field preparation<br>- Limited OR traffic<br>- Normothermia<br>- Euglycemia<br>- Wound protector<br>- Gown and glove change prior closure<br>- Closure trays<br>- Dressing removal within 48 hours<br>- Daily washing of incision with chlorhexidine (postoperative)
Kilan, 2017 [53]	- Antibiotic prophylaxis
Knox, 2016 [54]	- Antibiotic prophylaxis
Larochelle, 2011 [55]	- Antibiotic prophylaxis<br>- Normothermia
Lavu, 2012 [56]	- Smoking cessation<br>- Chlorhexidine-alcohol skin preparation (pre-admission)<br>- Hair removal<br>- Chlorhexidine-alcohol skin preparation<br>- Antibiotic prophylaxis<br>- Wound edge protection<br>- Glycemic control<br>- Temperature control<br>- Gown and glove change prior closure
Liau, 2010 [57]	- Hair removal<br>- Antibiotic prophylaxis<br>- Glucose monitoring<br>- Normothermia
Losh, 2017 [58]	- Smoking cessation<br>- Diabetic Screening<br>- Antibiotic bowel preparation<br>- Chlorhexidine wipes (Patient)<br>- Chlorhexidine<br>- Antibiotic prophylaxis<br>- Normothermia<br>- Glucose control<br>- Hair removal<br>- Glove change<br>- New instrument tray<br>- Wound protector<br>- Wound irrigation

First author, year	Clinical interventions (SSI preventive measures)
Lutfiyya, 2012 [59]	- Smoking cessation<br>- Antiseptic skin cleansing (chlorhexidine wipes, night before and morning of surgery)<br>- Mechanical colon preparation<br>- Antibiotic prophylaxis<br>- Diabetic Screening<br>- Glucose control<br>- Hair removal<br>- Normothermia<br>- skin preparation with chlorhexidine gluconate<br>- oxygen delivery<br>- double gloving<br>- pulse lavage of subcutaneous tissues (open operations)<br>- Protect primary-closure incisions with dressing for 5 days
Mammo, 2016 [60]	- Mechanical bowel preparation<br>- Antibiotic prophylaxis<br>- Heparin prophylaxis<br>- Normothermia
Misteli, 2012 [61]	- Antibiotic prophylaxis
Nordin, 2018 (Epub 2017) [62]	- Bowel preparation<br>- Abdomen cleansing (wipes, Patient)<br>- Normothermia<br>- Antibiotic prophylaxis<br>- Skin Prep with chlorhexidine or 10% povidone-iodine<br>- Glove change before closure<br>- Redrape surgical field<br>- Remove dirty instruments before closure<br>- Clean instruments for closure
Pastor, 2010 [63]	- Antibiotic prophylaxis<br>- Hair removal<br>- Glucose control<br>- Normothermia
Pérez-Blanco, 2015 [64]	- shower with chlorhexidine<br>- Glycaemic control<br>- Antibiotic Prophylaxis<br>- Normothermia<br>- Glove change<br>- Surgical wound dressing/cleansing
Reames, 2015 [65]	- Antibiotic prophylaxis<br>- Glucose control<br>- Hair removal<br>- Normothermia
Tanner, 2016 [66]	- Screening and decolonisation for MRSA<br>- Showering with soap<br>- Hair removal<br>- Antibiotic prophylaxis<br>- Skin preparation with chlorhexidine<br>- Normothermia<br>- Antiseptic impregnated incise drapes<br>- Supplemental oxygen<br>- Glucose control
Tillman, 2013 [67]	- Antibiotic prophylaxis<br>- Normothermia<br>- Hair removal
Vogel, 2010 [68]	- Antibiotic prophylaxis<br>- Wound closure/irrigation
Vu, 2018 (Epub 2017) [69]	- Antibiotic prophylaxis<br>- Mechanical bowel preparation<br>- Normoglycemia
Waters, 2017 [70]	- Antibiotic prophylaxis<br>(venous thromboembolism prophylaxis, urinary catheter removal)
Wick, 2012 [71]	- Skin preparation<br>- Chlorhexidine wash cloths<br>- Normothermia<br>- Sterile techniques (instruments, gloves)<br>- Antibiotic prophylaxis
Wick, 2015 [72]	- Smoking cessation<br>- Mechanical bowel preparation<br>- Chlorhexidine bathing<br>- Normothermia<br>- Antibiotic prophylaxis
Willis, 2016 [73]	- Antibiotic prophylaxis<br>- Local irrigation<br>- Drains
