# Supplementary material for: Implementation interventions in preventing surgical site infections in abdominal surgery: a systematic review
Source: BMC Health Serv Res. 2020 Mar 20;20:236. doi: 10.1186/s12913-020-4995-z (PMC7083020; doi:10.1186/s12913-020-4995-z)
Supplement: Supplementary file 3 — Additional file 3:Table S3. Quality assessment using the Quality Assessment Tool for Before-After (Pre-Post) Studies With No Control Group by the National Heart, Lung, and Blood Institute [31] [file 12913_2020_4995_MOESM3_ESM.pdf]

Additional file 3

Table S3: Quality assessment using the Quality Assessment Tool for Before-After (Pre-Post) Studies With No Control Group by the National Heart, Lung, and Blood Institute [31]

First author, year	1. Was the study question or objective clearly stated?	2. Were eligibility/ selection criteria for the study population pre-specified and clearly described?	3. Were the participants in the study representa-tive of those who would be eligible for the test/service/ intervention in the general or clinical population of interest?	4. Were all eligible participants that met the pre-specified entry criteria enrolled?	5. Was the sample size sufficiently large to provide confidence in the findings?	6. Was the test/service/ intervention clearly described and delivered consistently across the study population?	7. Were the outcome measures pre-specified, clearly defined, valid, reliable, and assessed consistently across all study participants?	8. Were the people assessing the outcomes blinded to the participants' exposures/ intervene-tions?	9. Was the loss to follow-up after baseline 20% or less? Were those lost to follow-up accounted for in the analysis?	10. Did the statistical methods examine changes in outcome measures from before to after the intervention? Were statistical tests done that provided p values for the pre-to-post changes?	11. Were outcome measures of interest taken multiple times before the intervention and multiple times after the intervention (i.e., did they use an interrupted time-series design)?	12. If the intervention was conducted at a group level (e.g., a whole hospital, a community, etc.) did the statistical analysis take into account the use of individual-level data to determine effects at the group level?	Total score (theoretical range: 0-12)
**Bull, 2011** [34]	1	1	0	1	NR	0	1	0	NR	0	1	1	6
**Cameron, 2015** [35]	1	1	0	CD	NR	1	1	NR	NR	1	0	1	6
**Cima, 2013** [36]	1	1	0	1	NR	1	1	NR	NR	1	1	1	8
**Connolly, 2016** [37]	1	1	0	1	NR	NR	1	NR	NR	1	0	1	6
**Crolla, 2012** [38]	1	1	0	1	0	1	1	NR	NR	1	0	1	7
**DeHaas, 2016** [39]	0	1	0	1	NR	NR	1	NR	NR	0	0	1	4
**Elia-Guedea, 2017** [40]	1	1	0	1	NR	NR	1	NR	NR	1	0	1	6
**Forbes, 2008** [41]	1	1	0	1	1	1	1	0	NR	1	0	1	8
**Frenette, 2016** [42]	1	1	0	1	1	NR	1	0	NR	1	0	1	7
**Garcell, 2017** [43]	1	1	0	1	NR	1	1	NR	NR	1	0	1	7
**Geubbels, 2004** [44]	1	1	1	CD	NR	NR	1	NR	NR	0	0	1	5
**Grant, 2018** (Epub 2017) [45]	1	1	0	1	NR	1	1	NR	NR	1	0	1	7
**Hechenbleikner, 2015** [46]	1	1	0	1	NR	1	1	NR	NR	1	1	1	8
**Hedrick, 2007** [47]	1	1	0	1	NR	1	1	NR	NR	1	0	1	7
**Hedrick, 2007** [48]	1	1	0	1	NR	1	1	NR	NR	1	0	1	7
**Hewitt, 2017** [49]	1	1	0	1	NR	NR	1	NR	NR	1	1	1	7
**Kao, 2010** [50]	1	1	1	1	NR	0	1	NR	NR	1	0	1	7
**Keenan, 2014** [51]	1	1	0	1	NR	1	1	NR	NR	1	0	1	7
**Keenan, 2015** [52]	1	1	0	1	NR	1	1	NR	NR	1	0	1	7
**Kilan, 2017** [53]	1	1	0	0	0	1	1	NR	NR	1	1	1	7
**Knox, 2016** [54]	1	1	0	1	1	0	1	NR	1	1	0	1	8
**Larochelle, 2011** [55]	1	1	0	1	NR	1	1	NR	NR	1	0	1	7
**Lavu, 2012** [56]	1	1	0	CD	NR	1	1	NR	NR	1	0	1	6
**Liau, 2010** [57]	1	1	0	1	1	1	1	NR	NR	1	0	1	8
**Losh, 2017** [58]	1	1	0	1	NR	1	1	NR	1	0	0	1	7
**Lutfiyya, 2012** [59]	1	1	0	1	0	NR	1	NR	NR	1	1	1	7
**Mammo, 2016** [60]	1	1	0	1	NR	1	1	NR	NR	1	0	1	7
**Misteli, 2012** [61]	1	1	0	1	1	0	0	NR	NR	1	0	1	6

First author, year	1. Was the study question or objective clearly stated?	2. Were eligibility/ selection criteria for the study population pre-specified and clearly described?	3. Were the participants in the study representa-tive of those who would be eligible for the test/service/ intervention in the general or clinical population of interest?	4. Were all eligible participants that met the pre-specified entry criteria enrolled?	5. Was the sample size sufficiently large to provide confidence in the findings?	6. Was the test/service/ intervention clearly described and delivered consistently across the study population?	7. Were the outcome measures pre-specified, clearly defined, valid, reliable, and assessed consistently across all study participants?	8. Were the people assessing the outcomes blinded to the participants' exposures/ intervene-tions?	9. Was the loss to follow-up after baseline 20% or less? Were those lost to follow-up accounted for in the analysis?	10. Did the statistical methods examine changes in outcome measures from before to after the intervention? Were statistical tests done that provided p values for the pre-to-post changes?	11. Were outcome measures of interest taken multiple times before the intervention and multiple times after the intervention (i.e., did they use an interrupted time-series design)?	12. If the intervention was conducted at a group level (e.g., a whole hospital, a community, etc.) did the statistical analysis take into account the use of individual-level data to determine effects at the group level?	Total score (theoretical range: 0-12)
**Nordin, 2018** (Epub 2017) [62]	1	1	0	1	NR	1	1	NR	0	1	0	1	7
**Pastor, 2010** [63]	1	1	0	1	NR	1	1	NR	NR	1	0	1	7
**Pérez-Blanco, 2015** [64]	1	1	0	1	NR	1	1	NR	NR	1	0	1	7
**Reames, 2015** [65]	1	1	1	1	1	NR	1	NR	NR	1	0	1	8
**Tanner, 2016** [66]	1	1	1	1	NR	1	1	0	1	1	0	1	9
**Tillman, 2013** [67]	1	1	0	CD	1	1	1	NR	NR	1	0	1	7
**Vogel, 2010** [68]	1	1	0	1	NR	1	1	NR	NR	1	0	1	7
**Vu, 2018** (Epub 2017) [69]	1	1	1	1	1	NR	1	NR	1	1	0	1	9
**Waters, 2017** [70]	1	1	0	1	1	1	1	NR	NR	1	0	1	8
**Wick, 2012** [71]	1	1	0	1	NR	1	1	NR	NR	1	1	1	8
**Wick, 2015** [72]	1	1	0	1	NR	NR	1	NR	NR	1	0	1	6
**Willis, 2016** [73]	1	1	0	1	0	1	1	NR	NR	1	0	1	7
**Total**	39	40	5	35	9	26	39	0	4	36	7	40	Mean: 7 SD: 0.96, 95%-confidence interval: [6.7│7.3]. range: 4-9)

Notes: Yes = 1; No = 0; CD = cannot determine; NA = not applicable; NR = not reported
