# Supplementary material for: Implementation interventions in preventing surgical site infections in abdominal surgery: a systematic review
Source: BMC Health Serv Res. 2020 Mar 20;20:236. doi: 10.1186/s12913-020-4995-z (PMC7083020; doi:10.1186/s12913-020-4995-z)
Supplement: Supplementary file 4 — Additional file 4:Table S4. Reporting of the implementation intervention audit and feedback [file 12913_2020_4995_MOESM4_ESM.pdf]

Additional file 4
Table S4: Reporting of the implementation intervention audit and feedback in N=32 studies

Description of “audit and feedback”	Studies			
	**Bull, 2011** [34]	**Cameron, 2015** [35]	**Cima, 2013** [36]	**Crolla, 2012** [38]
**Actors**	- project officer	NR	- audits: independent institutional observers<br>- feedback: the team	- infection control nurse, infection control practitioner<br>- multidisciplinary team: surgeons, anesthetists, head of the operating room, operating room personnel, infection control personnel
**Action**	- checklist accompanying each patient, allowing process compliance and outcome measures to be recorded<br>- compliance with bundle components was overseen<br>- focus groups to discuss progress<br>- clinician meetings: provide updates on documentation and progress of the project, and to reiterate the aims	- audit: antibiotic prescribed and time administered was recorded<br>- initial audit data were presented at a surgical mortality and morbidity meeting<br>- re-audited data were analyzed<br>- efficacy of the poster intervention was presented	- compliance audits: performance was monitored<br>- feedback was provided	- bundle adherence data were used to feedback /development of strategies for improvement<br>- bundle compliance was discussed in a multidisciplinary team<br>- newsletter provided feedback was distributed (results from compliance measurements, improvement recommendations)
**Action target group**	- compliance overseeing: clinical staff	- surgeons and anesthetists	- compliance audits: consultant surgeons, residents, nurses, allied health<br>- feedback: the team, staff	- newsletter: all personnel involved in surgical process
**Temporality**	NR	- audit: between October 2011/ January 2012<br>- re-audit: May 2012	NR	- adherence measurement: June 2009-October 2011
**Dose**	- focus groups: regular	- audit: 21 days<br>- re-audit: 28 days (after intervention)	- feedback: on a monthly basis	- bundle adherence measurement: every three months<br>- newsletter: every three months

Description of "audit and feedback"	Studies			
	**Elia-Guedea, 2017** [40]	**Forbes, 2008** [41]	**Frenette, 2016** [42]	**Garcell, 2017** [43]
**Actors**	NR	- working groups: surgeons, anesthesiologists, nurses, pharmacists from involved patient-care areas<br>- study champions: OR nurse, nurse from same-day surgery	- annual feedback: medical director of the infection prevention and control department; nursing consultant<br>- feedback after compliance lacking: infection prevention and control department<br>- compliance measurement: infection prevention and control department	compliance monitoring: infection-control practitioner<br>monitoring and presenting: pharmacist
**Action**	- clinical sessions in colorectal unit: information about detected problems and instruction on measures to be implemented	- practice audits<br>- performance data were collected for comparison<br>- three working groups to evaluate data from cohort I<br>- performance figures were posted in the OR to provide feedback<br>- study champions: inquired protocol implementation, provided direction, confirmed compliance	- annual feedback: individual SSI rates, overall SSI rates for each procedure, recommendations to decrease rates<br>- measuring compliance; if compliance was lacking: feedback and recommendations<br>- pilot audit and feedback project: feedback session, reminders to address identified deficiencies	- compliance monitoring with antibiotic prophylaxis<br>- analysis of department quality indicators<br>- feedback was provided and presented for analysis<br>- monitoring and presenting of antimicrobial consumption<br>- presentation of the antimicrobial stewardship program evaluation
**Action target group**	- clinical sessions: all surgeons of the colorectal unit	- performance figures: OR staff<br>- study champions: nurses, clinicians	- annual feedback: surgeons feedback after compliance lacking: surgical team<br>- feedback session: heads of surgical departments	- feedback: surgical team, facility infection control committee<br>- presentation of the evaluation: the staff
**Temporality**	NR	- first stage: (pre-intervention) collecting data on performance<br>- second stage: evaluate data<br>- final stage: (post-intervention) collecting data on performance, posting performance figures in the OR<br>- working group: meeting during a 1 year period (April 2005 - April 2006)	- began of annual feedback: June 2011<br>- began of compliance measurement and feedback: January 2011<br>- pilot audit and feedback project: October 2013<br>- second audit and feedback session: January - February 2014	NR
**Dose**	NR	- performance figures: monthly	- feedback: annual, after compliance lacking<br>- compliance measurement: periodically	- compliance monitoring: on an ongoing basis<br>- analysis of department quality indicators: monthly<br>- feedback: on monthly basis<br>- presentation of the evaluation: annual

Description of "audit and feedback"	Studies			
	**Geubbels, 2004** [44]	**Hechenbleikner, 2015** [46]	**Hedrick, 2007** [47]	**Hedrick, 2007** [48]
**Actors**	NR	chart review: two clinicians	NR	NR
**Action**	hospital E:<br>- monitoring of antibiotic prophylaxis and SSI incidence<br>- results were reported	- auditing tool to assess compliance<br>- retrospective chart review with the auditing tool<br>- identified defects were discussed<br>- meetings: addressing poor compliance and patient care defects	- feedback: compliance and infection rate	- monitoring outcomes measures<br>- individual feedback<br>- feedback: compliance and infection rate
**Action target group**	- result reporting: surgeons	- defect discussion: providers responsible for implementing measures	NR	- feedback: provider
**Temporality**	NR	NR	NR	- feedback: starts February 2005
**Dose**	- surveillance: ongoing<br>- result reporting: every 6 months	- meetings addressing poor compliance: weekly	- feedback: monthly	- feedback: monthly

Description of "audit and feedback"	Studies			
	**Kao, 2010** [50]	**Keenan, 2014** [51]	**Keenan, 2015** [52]	**Kilan, 2017** [53]
**Actors**	- compliance measures: trained research personnel	-meetings: colorectal surgeon (program leader)	- outcome abstraction: trained surgical-clinical reviewer<br>- overseeing implementation: colorectal surgeon<br>- meetings: colorectal surgeon, key personnel from each discipline<br>- colorectal surgery leadership meetings: colorectal surgery leadership, members of the multidisciplinary team	- feedback: quality improvement staff
**Action**	- compliance was measured<br>- extended timeout<br>-planned feedback	- meetings: review SSI results, address problems with bundle delivery<br>- audits of the bundle program	- outcome abstraction from medical record, conducting chart review<br>- obtaining compliance data from an institutional database<br>- overseeing implementation and program progress<br>- meetings: receive feedback on compliance, encourage compliance, review outcome data<br>- colorectal surgery leadership meetings: reinforce program objectives, identify problems, improve program coordination	- compliance feedback was given; reports that highlighted compliance<br>- personal reminders after non-compliance was identified
**Action target group**	- feedback: faculty	-meetings: designated key personnel	NR	- reminders: non-compliant providers<br>- feedback: the team
**Temporality**	- second study period (March-August 2008): intervention in hospital 1 (6 month)<br>- third study period (August 2008-November 2009): intervention in hospital 1 and 2 (6 month)	- audits: study period	NR	- feedback: implemented during April 2015 – July 2015<br>- reminders: implemented during April 2015 – July 2015
**Dose**	NR	- meetings: monthly	- meetings: monthly<br>- colorectal surgery leadership meetings: frequently	- feedback: regular / monthly<br>- reports: monthly

Description of "audit and feedback"	Studies			
	**Larochelle, 2011** [55]	**Liau, 2010** [57]	**Losh, 2017** [58]	**Lutfiyya, 2012** [59]
**Actors**	- performance monitoring: committees	- spread good results: hospital leadership	- preoperative compliance tracking: office staff member	NR
**Action**	- audit system with feedback<br>- performance was monitored<br>- compliance rates were made available	- compliance audit: tracking process measures and reporting findings<br>- visually reporting of updates in chart format<br>- good improvement results of the pilot population were spread	- SSI collaborative meetings compliance was tracked, presented, discussed<br>- SSI root cause analysis: monitoring breakdown of implemented measures<br>- OR meetings: sharing SSI rates, track and encourage compliance, discuss strategies to improve adherence and safety culture	- reviewing process measures and SSI cases, addressing gaps<br>- calculating SSI rate, developing a run chart, establishing reports<br>- SSI Quality Group meetings: data tracking<br>- departmental meetings: reviewing outcomes<br>- a report was provided if a measure was omitted<br>- individual feedback was provided if defects were identified<br>- posting SSI dashboards in surgeon and operating room lounges<br>- monitored components were posted in surgical and physicians lounges<br>- reporting performance comparison with peers
**Action target group**	NR	- reporting: the staff<br>- updates in chart format: all staff in the OR, post-anesthesia care unit, wards<br>- spread good results: to other surgical disciplines	- OR meetings: surgery staff	- SSI Quality Group meetings: all appropriate care providers<br>- reporting of peer comparison: all involved stakeholders: administrators, surgeons, nursing staff, infection control, SSI Quality Committee<br>- standardized report: surgeons<br>- individual feedback: groups of providers
**Temporality**	- compliance rates were made available: starting in 2004	NR	NR	NR
**Dose**	- compliance rates were made available: quarterly	- tracking process measures: on a monthly basis<br>- reporting: regularly<br>- updates in chart format: frequently	- SSI collaborative meetings: monthly<br>- OR meetings: monthly<br>- chart reviews: quarterly	- SSI rate calculation: every month<br>- reports: quarterly<br>- review of SSI/process measures: on a monthly basis /regular basis<br>- SSI Quality Group's meetings: monthly

Description of "audit and feedback"	Studies			
	**Nordin, 2018 (Epub 2017)** [62]	**Pastor, 2010** [63]	**Pérez-Blanco, 2015** [64]	**Reames, 2015** [65]
**Actors**	NR	recording compliance: infection control professional<br>monitoring: multidisciplinary task force (surgeons, anesthesiologists, infection control personnel, intraoperative nurses)	NR	- compliance monitoring: operative teams
**Action**	- monitoring and analyzing bundle compliance<br>- feedback was provided	- compliance was recorded<br>- individual compliance rates and infections were collected and reviewed<br>– meetings: compliance monitoring and addressing barriers	- collecting variables related to adherence<br>- evaluating adherence to protocol<br>- commitment to provide feedback on results	- outcome measurement and feedback<br>- compliance monitoring<br>- routine briefings and debriefings<br>- content and coaching calls<br>- semiannual daylong collaborative meetings
**Action target group**	NR	NR	NR	- content and coaching calls / collaborative meetings: operative team leaders (in each hospital: surgeon, anesthesiologist, operating room nurse)<br>- briefings and debriefings: surgical teams
**Temporality**	- monitoring and analyzing bundle compliance: start in August 2014	- multidisciplinary task force: April 2006	NR	NR
**Dose**	- monitoring and analyzing bundle compliance: on a monthly basis<br>- feedback: ongoing	- recording compliance: on a weekly basis<br>- meetings: monthly<br>- review of compliance rates and infections: monthly	- feedback: at least biannually	- content and coaching calls: monthly<br>- collaborative meetings: semiannual daylong

Description of "audit and feedback"	Studies			
	**Tanner, 2016**[66]	**Tillman, 2013** [67]	**Vogel, 2010** [68]	**Vu, 2018 (Epub 2017)** [69]
**Actors**	- compliance measurement: project assistant<br>- SSI measurement: surveillance team	monitoring: multidisciplinary team	NR	site visits: project leaders, committed clinicians<br>progress discussion: members from participating hospitals
**Action**	- compliance with interventions and SSIs were measured<br>- feedback: compliance data and SSI rates were posted in key areas (e.g. scrub rooms)	- monitoring and coaching<br>- collecting and reporting compliance and outcome data	- outcomes were recorded<br>- morbidity and mortality (M+M) conferences: discussing data, developing a new strategy and a benchmark was carried out<br>- reporting annual results in an conference<br>- implementation of the new strategy was reviewed	- audit and feedback system for adherence<br>- measuring compliance and SSI rates<br>- auditing system: providing de-identified feedback on personal SSI bundle compliance/SSI rates<br>- assigning bundle compliance scores to hospitals<br>- compliance tracking allows: normative performance feedback compared with other hospitals<br>- site visits: observing perioperative care processes (process adoption real time checks)<br>- discussing progress and refining new ideas
**Action target group**	NR	- monitoring and coaching: surgical teams<br>- reporting compliance and outcome data: regulatory bodies	NR	- auditing system: all providers<br>- feedback: surgeons, hospitals<br>- site visits: high-and low-performers
**Temporality**	NR	NR	NR	NR
**Dose**	- feedback: monthly	NR	- M+M conferences: 1-2 monthly<br>- conference: annual	- site visits: six over study period<br>- progress discussion: frequently

Description of "audit and feedback"	Studies			
	**Waters, 2017** [70]	**Wick, 2012** [71]	**Wick, 2015** [72]	**Willis, 2016** [73]
**Actors**	- data collection: trained unbiased nurse/ physician's assistant	- meetings: Comprehensive Unit-Based Safety Program (CUSP) leadership team (representative from surgery, nursing, anesthesia; team coach; hospital executive) and the CUSP team (interested nurses, certified registered nurse anesthetists, scrub technicians, anesthesiologists; 36 people)<br>- compliance monitoring: CUSP coach<br>- feedback: front-line providers	- monitoring: senior leaders	NR
**Action**	- data collection on all procedures<br>-surgeon-specific feedback: adherence, outcomes<br>- individualized feedback and feedback at department level<br>- individual results, and comparison with: other surgeons results, departmental results, historical departmental results	- conducting compliance audits<br>- collection of SSI rates and compliance<br>- meetings: review progress, give performance feedback, conduct defect discussions, readjust implementation strategies	- monitoring results<br>- compliance data were reviewed<br>- dashboard: communicating pathway process and outcomes measures	- calculating and assessing adherence rate<br>- performance reports: overall and individual adherence
**Action target group**	- feedback: eight surgeons (colon and rectal)	NR	- compliance data review: providers<br>- dashboard: senior leadership, frontline providers	- performance reports: pediatric surgeons
**Temporality**	NR	NR	NR	NR
**Dose**	- feedback: biannual	- compliance audits: every few month<br>- meetings/feedback: monthly	- dashboard: monthly	- performance reports: monthly

Notes: NR: Not reported
